# The Effect of Oral Vitamin E on Semen Parameters and IVF Outcome: A Double-Blinded Randomized Placebo-Controlled Clinical Trial

**DOI:** 10.1155/2021/5588275

**Published:** 2021-10-11

**Authors:** Soudabeh Sabetian, Bahia Namavar Jahromi, Sina Vakili, Sedighe Forouhari, Shohreh Alipour

**Affiliations:** ^1^Infertility Research Center, Shiraz University of Medical Sciences, Shiraz, Iran; ^2^Department of Obstetrics & Gynecology, School of Medicine, Shiraz University of Medical Sciences, Shiraz, Iran; ^3^Pharmaceutical Sciences Research Center, School of Pharmacy, Shiraz University of Medical Sciences, Shiraz, Iran; ^4^Department of Quality Control, School of Pharmacy, Shiraz University of Medical Sciences, Shiraz, Iran

## Abstract

**Background:**

Male infertility is a main clinical problem that affects about 7% of all men worldwide. Many patients with male infertility are caused by a reduced antioxidant capacity of semen. Several antioxidant supplements, especially vitamin E, are proposed to help male infertility treatment. This project was goaled to study the effects of oral synthetic vitamin E (400 IU/day) for eight weeks on betterment of semen parameters and pregnancy rate.

**Methods:**

After dropping the cases, 124 infertile couples with a male factor who were admitted to the IVF program were included. The male patients with idiopathic abnormal motility and/or morphology were randomized into two groups: 61 receiving vitamin E and 63 as the control group receiving placebo for eight weeks. The pretreatment semen parameters of both groups were compared with those of posttreatment. The pregnancy outcomes were considered between the two groups.

**Results:**

There were no significant differences statistically between before and after treatment in the term of sperm volume, count, motility, and morphology. Furthermore, the IVF outcomes of the two groups were not different significantly, either. Interestingly, the percent of normal sperm in the placebo group was significantly decreased after eight weeks.

**Conclusion:**

Vitamin E supplementation might neutralize free radical activity to keep sperm from more oxidative damages. Further studies regarding the influence of higher acceptable doses of vitamin E on semen characteristics and fertility rates are needed. This study was registered as a two-arm, blinded, randomized, placebo-controlled clinical trial (IRCTID: IRCT2014020616506N1, 2014-03-18).

## 1. Background

Infertility is a significant clinical problem, affecting about 15% of all couples at reproductive age [1]. In around half of the infertile couples, a male factor is the only identifiable cause [2].

Decreased function and quality of sperm lead to decreased male fertilization potential. Evaluation of semen parameters and quality is the first laboratory step for assessing male fertility problems [3].

Some factors such as infection, environmental, and genetic factors are associated with semen quality; however, the implicated etiology is poorly understood [4]. Although several nutrient supplementation products are promised to improve semen parameters, there are a few available evidence-based data regarding nutrient effects on the treatment of male infertility [5]. The environmental factors such as air pollution, electromagnetic radiation, and poor nutrition cause to amplify reactive oxygen species (ROS) and subsequent oxidative stress [6]. Previous researchers showed that ROS and oxidative stress reduce male fertility [7]. Sperm plasma membrane consists of a large amount of polyunsaturated fatty acids that are assailable by ROS [8]. Lipid peroxidation mechanism seriously impresses on membrane function and subsequently decreases sperm quality [9]. An extreme level of ROS can damage sperm DNA through the stimulation of sperm caspases and endonucleases [10].

Antioxidants including vitamin E, superoxide dismutase, vitamin C, thioredoxin, and glutathione can neutralize free radical activity to keep sperm from produced ROS and subsequently improve male fertility [11]. Many studies have revealed a strong positive relevance between antioxidant consumption and improved male infertility [12].

Previous researchers have stated that the male supplementation by antioxidants such as vitamins E and C before intracytoplasmic sperm injection (ICSI) and in vitro fertilization (IVF) decreased the level of DNA damage and therefore enhanced pregnancy success rates [13]. However, some efforts have explored paradoxical results [14].

The purpose of this project was to study if consumption of 400 IU synthetic vitamin E (*α*-tocopherol) daily for eight weeks can affect semen parameters or pregnancy outcomes in couples with male factor infertility.

## 2. Materials and Methods

### 2.1. Subjects

**Figure 1:**
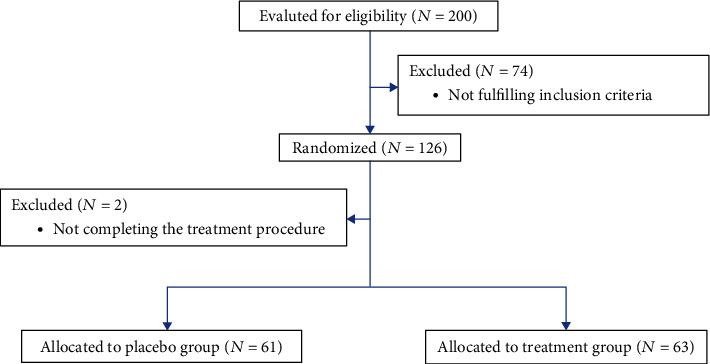
CONSORT flowchart of study participants.

Two-hundred couples who were referred for IVF at Ghadir Mother and Child Hospital infertility center with male factor infertility (primary or secondary) were the candidates to be enrolled in this study. The couples had not conceived after at least a one year of regular attempts for pregnancy without female factor infertility. The included men aged 20-45 years, and male factor infertility was diagnosed according to WHO criteria. 74 men with an allergy to vitamin E or they were not willing to participate or did not comply the inclusion criteria were not enrolled in the study. Therefore, 126 men were randomly divided into two groups using a block-randomization list. Two cases did not continue the medication and were excluded (Figure 1). All couples were involved in the IVF process.

### 2.2. Study Design

The study process was planned as a two-arm, blinded, randomized, placebo-controlled clinical trial (IRCTID: IRCT2014020616506N1, 2014-03-18, https://fa.irct.ir/trial/15426).

### 2.3. Ethical Issues

All the patients who complied the inclusion criteria of the study signed the written informed consent before enrolment according to the Declaration of Helsinki (1989 revision). Local Medical Ethics Committee of Shiraz University of Medical Sciences agreed with the study protocol with the reference number CT-P-92-5604.

### 2.4. Intervention

Coded plastic packs each containing 60 capsules that were prepared by the pharmaceutics department of SUMS were given to the patients. The patients were instructed to take one pill daily for eight weeks. The pills contained either 400 IU synthetic vitamin E (*α*-tocopherol) or placebo with similar shapes. A routine lifestyle was recommended for the patients. To monitor their compliance, all participants were requested to bring their capsule packages for every month's visits.

### 2.5. Statistical Analysis

The sample size was calculated to be 60 participants in each group by considering a one-sided significance level of 0.05, power of 0.80, and dropout rate of 20%.

To analyze the outcomes, the intention-to-treat analysis protocol was used for the patients who did not completely meet the study protocol. Clinical characteristics of the patients were shown by mean ± standard deviation for numerical percent for categorical variables. The statistical analyses included *T*-test and chi-square for numerical and categorical outcomes, respectively. To measure how the patients fare before and after the treatment, paired *T*-test was applied. *P* values <0.05 were remarked significant. The Statistical Package for the Social Sciences, version 19.0 (SPSS Inc. Chicago, IL, USA) was used for the current statistical analyses.

## 3. Results

Of 126 patients who enrolled in the study, only two subjects in the placebo group withdrew from the study because of missed follow-up. A total of 124 patients completed the study protocols that 104 cases (≈84%) had primary infertility and 20 cases (≈16%) suffered from secondary infertility. Demographic data of the cases are displayed in Table 1.

61 patients received placebo, and 63 cases received vitamin E. The age of the patient and their female partners and also semen parameters at the beginning of the study are presented in Table 2. All of the participants had abnormal semen parameters. There were no significant differences between the two groups.

During the 8-week treatment period, supplementation with 400 IU daily synthetic vitamin E (*α*-tocopherol) resulted in no significant differences in semen parameters (Table 3). However, the number of sperms with normal morphology was reduced significantly after eight weeks in the patients who received placebo (Table 3).

After treatment, the couples underwent IVF with fresh embryo transfer, and the pregnancy results were investigated between placebo and vitamin E groups. The pregnancy rate of the cases is shown in Table 4. A total of 15 patients (12.1%) gave birth to a live baby. The rate of pregnancy and living birth was not significantly different between the placebo and vitamin E groups (Table 4).

## 4. Discussion

This study is aimed at considering the influence of fixed-dose 400 IU synthetic vitamin E on semen parameters of men who have infertility. We compared the differences in sperm concentration, morphology, and motility as well as pregnancy rate. Live birth rates between the groups that had oral administration of vitamin E supplement were compared to those who took the placebo and found no significant difference between them.

Several studies represented a significant positive impact of antioxidant supplements on semen characteristics, pregnancy, and success rates for assisted reproductive technology (ART) [15]. Vitamin E, vitamin C, zinc, folic acid, coenzyme Q10, selenium, l-carnitines, *N*-acetyl cysteine, and lycopene are the most commonly prescribed. In most of the cases, the main cause of male factor infertility at the molecular level remains unknown [16]. Previous efforts have highlighted a significant contribution of oxidative stress, caused by too high amounts of oxidants or too low levels of antioxidants, in idiopathic infertility [17].

Free radicals or ROS are highly reactive oxygen-derived molecules that are produced by sperm cells in small amount and play an essential role in sperm capacitation, sperm maturation, and cellular signaling pathway [18–21]. An excessive amount of ROS causes increase lipid peroxidation and DNA damage in the sperm cell, ultimately disruptive effect on sperm function leads to infertility [22]. About 25% of infertile men were demonstrated to have a high ROS level in their semen [23]. Recent reports have indicated a significant negative impression of ROS on semen parameters, fertilization, embryo development, and pregnancy rate [24]. Therefore, the reduction of ROS through antioxidant treatment seems to be a treatment strategy to help pregnancy naturally or by ART. However, a clear consensus regarding the clinical success of treatment by antioxidant is still debatable.

A systematic review evaluated the efficacy of various micronutrient supplements, alone or in combination on semen parameters as well as pregnancy rate. Two studies were considered the impact of antioxidant compounds on the semen characteristics [25]. In one of the studies, high-dose vitamin C (1000 mg/day) in combination with vitamin E (800 mg/day) was administered to men with asthenozoospermia or moderate oligoasthenozoospermia [26]. The other study prescribed only vitamin E (600 mg/day) to men with high amounts of ROS in their sperm [27]. No significant improvement could be displayed in sperm motility, morphology, and concentration as well as pregnancy rate applying treatment by antioxidant compared with placebo in the two studies. Tremellen and his colleagues administrated an antioxidant supplement consisting of vitamin C, vitamin E, zinc, folic acid, lycopene, garlic oil, and Se to couples with severe infertility per day for three months before the IVF cycle [28]. They observed a significant improvement in the pregnancy rate (38.5%) compared to a placebo group (16%). Since 1 mg of alpha-tocopherol is equivalent to 2.2 IU of the synthetic form, the patients took about 181.82 mg of vitamin E per day in our study.

The results of our study showed no significant betterment in sperm parameters and pregnancy rate following oral daily vitamin (181.82 mg/day) for eight weeks compared with placebo. Spermatids elongate to form spermatozoa during spermiogenesis and are evacuated from the Sertoli cells to epididymis for final maturation. Spermatozoa in epididymis obtain motility and acrosomal function, which are essential for successful fertilization [29]. The full cycle for sperm development from production to ejaculation of new mature spermatozoa takes average of 64 days to complete (with a range of 42-76 days) [30, 31]. The required time for the sperm production process will vary depending on the individual's genetic, lifestyle, and age [32]. The reason for the absent significant correlation between consumption of 400 IU vitamin E and improvement in semen parameters and IVF/ICSI success rate might be because our subjects received a low dose of vitamin E and only for eight weeks. We know the strength of our study that was designed as a double-blinded placebo-controlled RCT, and also, we see the limitation of our research that vitamin E (181.82 mg/day) with a low dose and only for eight weeks was used.

## 5. Conclusion

In the present study, there were no significant differences in semen parameters and fertility rates in infertile males by intaking vitamin E (400 IU/day, for eight weeks). Interestingly, a substantial reduction of normal sperms was observed in the placebo group after eight weeks. It is speculated that vitamin E might delay the progression of oxidative damages to sperm. Additional researches are also needed to evaluate the efficacy of safe upper limits of vitamin E for a more extended period on semen parameters and IVF outcome in infertile couples with male factor.

## Figures and Tables

**Table 1:** Demographic data.

Cases with primary infertility	104
Cases with secondary infertility	20
Male age (mean ± SD)	36.11 ± 6.88
Female partner age (mean ± SD)	31.50 ± 5.64

**Table 2:** Age and baseline semen parameters of vitamin E and placebo groups.

Variables	Placebo treatment	Vitamin E treatment	*P* value
Patient age (years)	35.76 ± 7.24	36.72 ± 6.61	0.60
Female partner age (years)	31.28 ± 6.00	31.71 ± 5.27	0.67
Sperm volume (ml)	3.45 ± 1.59	3.11 ± 1.61	0.25
Sperm concentration (×10^6^/ml)	9.91 ± 10.92	12.16 ± 16.21	0.38
Sperm motility (% WHO grade *a* + *b*)	32.27 ± 13.93	31.08 ± 13.27	0.63
Sperm morphology (% normal)	2.37 ± 1.57	2.22 ± 1.66	0.36

Results are represented as mean ± SD.

**Table 3:** Semen parameters in placebo and vitamin E group before and after trial (paired *T*-test).

Groups	Mean ± SD	Paired differences			
		Mean ± SD	95% confidence interval of the difference		*P* value
			Lower	Upper	
Vitamin E					
Volume 1 (ml)	3.11 ± 1.61	−.011 ± 1.82	-0.47	0.44	0.96
Volume 2 (ml)	3.12 ± 1.37				
Count 1 (×10^6^/ml)	12.16 ± 16.21	2.10 ± 8.93	-0.14	4.35	0.06
Count 2	10.05 ± 13.34				
Motility 1 (% WHO grade *a* + *b*)	31.08 ± 13.27	−0.71 ± 15.75	-4.68	3.25	0.72
Motility 2 (% WHO grade *a* + *b*)	31.80 ± 14.29				
Morphology 1 (% normal)	2.22 ± 1.66	0.36 ± 1.65	-0.05	0.78	0.08
Morphology2 (% normal)	1.85 ± 1.35				
Placebo group					
Volume 1) ml)	3.34 ± 1.59	0.19 ± 1.71	-0.24	0.64	0.37
Volume 2) ml)	3.25 ± 1.41				
Count 1 (×10^6^/ml)	9.91 ± 10.92	0.01 ± 6.50	-1.65	1.67	0.98
Count 2 (×10^6^/ml)	9.90 ± 14.79				
Motility 1 (% WHO grade *a* + *b*)	32.27 ± 13.93	0.42 ± 13.09	-2.92	3.77	0.80
Motility 2 (% WHO grade *a* + *b*)	31.85 ± 12.86				
Morphology 1 (% normal)	2.37 ± 1.57	0.40 ± 1.64	-0.01	0.83	0.05
Morphology 2 (% normal)	1.97 ± 1.33				

1: before treatment and 2: after treatment.

**Table 4:** IVF results of the placebo and vitamin E groups after treatment period.

IVF results	Placebo (*n* = 61)	Vitamin E (*n* = 63)	*P* value
Cancellation of cycles	6 (9.8%)	10 (16.4%)	0.41
No embryo developed	8 (13.1%)	5 (8.2%)	0.55
Negative clinical-chemical pregnancy	35 (57.4%)	38 (62.3%)	0.70
Positive chemical pregnancy	12 (19.7%)	10 (16.4%)	0.80
Abortion before 14 weeks	2 (3.3%)	4 (6.6%)	0.66
Abortion after 14 weeks	1 (1.6%)	0	0.99
Live birth	9 (14.8%)	6 (9.8%)	0.56

## Data Availability

The datasets used and analyzed during the current study are available from the corresponding author on reasonable request.

## Abbreviations

ICSI: Intracytoplasmic sperm injection
IVF: In vitro fertilization.
